# A Novel Graph Neural Network Method for Traffic State Estimation with Directional Wave Awareness

**DOI:** 10.3390/s26010289

**Published:** 2026-01-02

**Authors:** Xiwen Lou, Jingu Mou, Boning Wang, Zhengfeng Huang, Hang Yang, Yibing Wang, Hongzhao Dong, Markos Papageorgiou, Pengjun Zheng

**Affiliations:** 1 Faculty of Maritime and Transportation, Ningbo University, Ningbo 315832, China; 2311120008@nbu.edu.cn (X.L.); 2311120121@nbu.edu.cn (J.M.); huangzhengfeng@nbu.edu.cn (Z.H.); yanghang@nbu.edu.cn (H.Y.); mpapageorgiou@tuc.gr (M.P.); 2 School of Economics, The University of Edinburgh, Edinburgh EH8 9YL, UK; s2607903@ed.ac.uk; 3 Collaborative Innovation Center of Modern Urban Traffic Technologies, Southeast University, Nanjing 211189, China; 4 National Traffic Management Engineering & Technology Research Centre, Ningbo University Sub-Centre, Ningbo 315832, China; 5 Institute of Intelligent Transportation Systems, Zhejiang University, Hangzhou 310058, China; wangyibing@zju.edu.cn; 6 School of Mechanical Engineering, Zhejiang University of Technology, Hangzhou 310023, China; its@zjut.edu.cn; 7 Dynamic Systems and Simulation Laboratory, Technical University of Crete, 73100 Chania, Greece

**Keywords:** traffic state estimation, graph neural network, kriging, traffic wave theory, fundamental diagram, spatiotemporal correlation

## Abstract

Traffic state estimation (TSE) is crucial for intelligent transportation systems, as it provides unobserved parameters for traffic management and control. In this paper, we propose a novel physics-guided graph neural network for TSE that integrates traffic flow theory into an estimation framework. First, we constructed wave-informed anisotropic temporal graphs to capture the time-delayed correlations across the road network, which were then merged with spatial graphs into a unified spatiotemporal structure for subsequent graph convolution operations. Then, we designed a four-layer diffusion graph convolutional network. Each layer is enhanced with squeeze-and-excitation attention mechanism to adaptively capture dynamic directional correlations. Furthermore, we introduced the fundamental diagram equation into the loss function, which guided the model toward physically consistent estimations. Experimental evaluations on a real-world highway dataset demonstrated that the proposed model achieved a higher accuracy than benchmark methods, confirming its effectiveness in capturing complex traffic dynamics.

## 1. Introduction

With the rapid advancement of urbanization, intelligent transportation systems (ITSs) have emerged to analyze and improve traffic conditions. As an essential part of ITSs, traffic state estimation (TSE) plays a critical role in inferring traffic state variables (e.g., flow, speed, density) from partially observed data [1,2]. However, accurate estimation remains challenging due to sparse sensor distributions [3], a limited data collection frequency, and transmission noise [4]. As traffic control is generally implemented on a network-wide scale, there is a pressing need for efficient and accurate methods that enhance the spatial resolution of available traffic data. To address this need, our study focuses on network-wide highway TSE, aiming to reconstruct real-time traffic states across entire sensor networks using spatially sparse measurements.

The existing freeway TSE approaches are typically classified into three categories, namely model-driven, data-driven, and hybrid physics-based machine learning methods. Model-driven methods integrate traffic flow theory with mathematical models to determine the traffic state, typically utilizing macroscopic traffic flow models like the first-order Lighthill–Whitham–Richards (LWR) model [5,6] and high-order models such as METANET [7]. These models are computationally efficient and highly interpretable, but require extensive parameter calibration, and often lack adaptability under complex real-world conditions. Data-driven approaches utilize statistical and machine learning techniques to exploit historical traffic data, enabling flexible adaptation to diverse traffic scenarios. Their effectiveness, however, is constrained by the availability and quality of large-scale datasets, and their black-box nature limits their interpretability, making it difficult to understand their underlying estimation mechanisms. Both approaches, therefore, face significant challenges when data are sparse and noisy.

To overcome these limitations, hybrid physics-based machine learning methods have emerged, combining established traffic flow theory with adaptive learning techniques. Such approaches utilize the strengths of both physics-based principles and the representational power of deep learning, providing a promising balance between realism and flexibility.

In this paper, we propose a hybrid physics-based graph neural network (GNN) method for TSE that explicitly embeds traffic flow theory. A temporal graph, derived from kinematic wave theory, was constructed to model time-delayed and anisotropic correlations across the road segments. Then, spatial and temporal graphs were integrated as an untied spatiotemporal graph, which was then incorporated into graph convolution operations. An improved GNN model was designed to capture dynamic spatial and temporal dependencies. Additionally, the FD was incorporated as a physics-based constraint in the loss function, guiding training toward physically consistent estimates. In summary, our contributions are as follows:

(1) We constructed anisotropic temporal graphs guided by traffic wave propagation and merged them into a novel spatiotemporal fusion graph for GNN, which effectively shows the time-delayed correlations between road segments and outperforms only-conventional spatial-distance-based graphs.
(2) We designed a deep GNN framework, where each layer is a diffusion graph convolutional network enhanced with a squeeze-and-excitation attention mechanism. This architecture enables dynamic and directional dependency capture across the road network.
(3) We introduced the fundamental diagram equation as a physics-based constraints into the loss function, ensuring physical consistency and improving the robustness.
(4) Experimental results using real-world highway data showed that the proposed model achieved a superior performance compared to both conventional methods and purely data-driven deep learning baselines, underscoring the value of integrating domain-specific traffic flow theory into deep learning frameworks for more accurate and reliable traffic state estimation.

The rest of this article is organized as follows. Section 2 reviews related work on model-driven, data-driven, and hybrid physics-based machine learning approaches for TSE. Section 3 provides basic concepts and formally defines the problem. Section 4 details the proposed model architecture and explains how domain knowledge of traffic flow was integrated into the GNN. Section 5 presents experimental results and ablation studies conducted on a real-world traffic dataset. Section 6 concludes the paper and outlines potential future research directions.

## 2. Literature Review

Numerous studies have investigated traffic state estimation (TSE), and the existing methods can be broadly categorized into three groups: model-driven, data driven, and hybrid physics-based machine learning approaches. This section reviews each in turn.

### 2.1. Model-Driven Methods of TSE

Model-driven methods estimate and predict traffic states based on traffic flow models derived from theoretical frameworks such as conservation laws and fluid dynamics. Commonly applied frameworks include the LWR model [5,6] and the METANET model [7], which describe the fundamental physical principles of traffic flow.

Within this category, filtering approaches are widely used to combine macroscopic traffic flow models with measurement models in a state-space representation. The Kalman filter (KF) was among the earliest methods applied for TSE and remains popular due to its computational efficiency and low memory requirements. However, the KF is limited to linear systems. To overcome this, the extended Kalman filter (EKF) incorporates nonlinear models, but requires differentiability [8,9,10]. The unscented Kalman filter (UKF) eliminates this requirement, allowing for nonlinear systems without explicit derivatives [3,11,12,13]. Particle filters (PFs) further extend the applicability by estimating posterior distributions through Monte Carlo resampling [14,15,16]. Despite their strengths, model-driven approaches depend heavily on accurate parameter calibration, and their performance deteriorates when the model parameters deviate from real-world traffic conditions.

### 2.2. Data-Driven Methods of TSE

Data-driven approaches directly estimate unobserved traffic variables using historical data without explicit flow models. With the advent of machine learning, more sophisticated methods emerged. Cheng et al. improved fuzzy c-means clustering by refining membership functions and sample weighting [17]. Babu et al. introduced sparse Bayesian learning (SBL) and block SBL (BSBL) for sparse representations [18], while Wu et al. applied Gaussian processes with anisotropic kernels to model congestion propagation [19].

Deep learning has further expanded TSE capabilities, employing convolutional neural networks (CNNs) [20], generative adversarial networks (GANs) [21,22], transfer learning (TL) [23], and diffusion models [24]. Graph neural networks (GNNs) have become especially influential in capturing spatial and temporal correlations [25,26,27,28,29,30]. Wu et al. proposed an adaptive graph learning module to generalize diffusion processes [31]. Lin et al. proposed a novel spatiotemporal GNN to predict traffic congestion [32]. Odiagbe et al. proposed an enhanced GNN to provide more accurate traffic forecasts than traditional GNNs [33]. These advances highlight the power of GNNs for network modeling. Importantly, while prediction tasks rely on abundant historical data to forecast future states, TSE typically aims to infer the current conditions at unmeasured locations, a task more constrained by data availability.

Recent work has also connected TSE to geostatistics, where unobserved values are estimated via spatial kriging. Wu et al. introduced a kriging convolutional network to estimate the spatiotemporal traffic speed using GNNs [25], with subsequent improvements from Liang et al. [27] and Nie et al. [28]. These studies reflect the value of the kernel design for capturing spatial correlations in traffic networks. Although these studies powerfully modeled spatial dependencies, they primarily operated within a data-driven paradigm.

### 2.3. Hybrid Physics-Based Machine Learning Methods of TSE

Hybrid methods combine the interpretability of physics-based models with the adaptability of machine learning. Their improvements generally stem from two directions: data augmentation and the structural integration of physical models.

Unlike conventional methods, these hybrid methods utilize traffic physics to improve the estimation accuracy and precision. Performance improvements in hybrid methods primarily come from two aspects: data augmentation and the structural integration of physical models [34]. For data augmentation methods, Zhang et al. augmented training datasets with traffic flow simulations to enhance machine learning models [35]. While more recent approaches have embedded physics directly into learning architectures, researchers have integrated traffic flow models into loss functions. Shi et al. proposed physics-informed deep learning with a fundamental diagram (FD) learner to encode physical complexity while maintaining trainability [36,37]. Huang and Agarwal integrated the LWR and cell transmission models into loss functions to address sparse and noisy data [38]. Pereira et al. embedded macroscopic flow discretization into recursive neural networks [39]. Zhang et al. used computational graph methods to infer FD parameters [40]. Then, researchers tried to integrate physics law into more complex deep learning structures. Thodi et al. incorporated kinematic wave theory into a convolutional network for estimating high-resolution velocity fields [41]. Pan et al. developed an FD–Markov–LSTM model that combined traffic physics with temporal sequence modeling [42].

Given the significant complexity involved in directly embedding full traffic flow models into GNN architectures, our work diverged from this approach. Instead, we introduced a novel hybrid framework that imposes physics-based constraints through an alternative mechanism for TSE. It models spatiotemporal correlations among road segments using kinematic wave theory, employs a diffusion graph convolutional network (DGCN) to estimate traffic states, and incorporates the FD as a physics-based constraint within the loss function. Experiments with real-world data confirmed that the proposed approach significantly improved the estimation accuracy, providing valuable insights for network-wide traffic control and management.

## 3. Preliminaries and Problem Descriptions

In this section, we introduce the foundational concepts, describe the traffic parameter estimation problem, and explain how traffic state estimation (TSE) can be formulated as a kriging problem within a graph neural network (GNN) framework.

### 3.1. Graph Representation of Traffic Network

**Figure 1:**
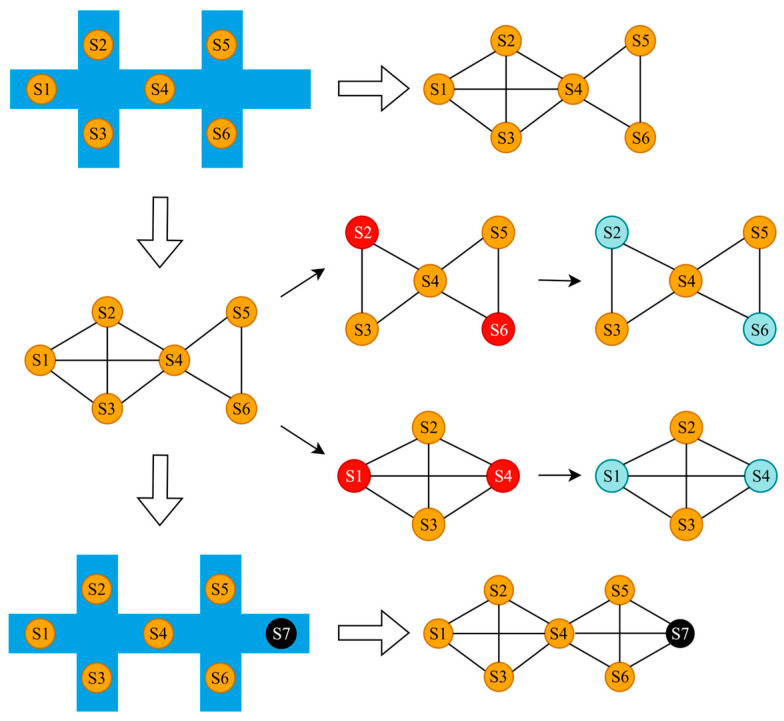
Explanation of the graph construction and the operation of the kriging model. Orange dots are sensors on the road. Black dots denote the points to be estimated. Red dots are assumed unknown points in training and blue dots denote their completed counterparts.

Network-wide TSE is a spatiotemporal sequence interpolation problem that seeks to reconstruct traffic states across unobserved road segments using sparse sensor data. This task is naturally formulated on graph representations of road networks, where topological constraints govern state propagation. As illustrated in Figure 1, we modeled the road network as a weighted and directed spatial graph structure $G=\left(V,E,A\right)$, where $V=\left\{v_{i}\right\}$ denotes sensor locations, $E=\left\{e_{ij}\right\}\;$ represents physical connections between nodes, and $A\in R^{N\times N}$ is the weighted adjacency matrix encoding edge weights.

To account for the decay in the interaction strength with distance, adjacency matrix A is commonly defined using a Gaussian kernel function with a threshold [43], as shown in Equation (1):

(1) $$a_{ij}=\left\{\begin{array}{c} \exp\left(-{\left(\frac{dist\left(v_{i},v_{j}\right)}{\delta }\right)}^{2}\right),\;if\;dist\left(v_{i},\;v_{j}\right)\leq \epsilon ,\\ 0,\;otherwise \end{array}\right.$$

where $a_{ij}$ encodes the connection between the upstream node $v_{i}$ and the downstream one $v_{j}$, dist(·) denotes the travel distance from $v_{i}$ to $v_{j}$, δ is the standard deviation of the distances, and ϵ is the threshold. This kernel ensures that $a_{ij}\;\approx \;1$ when the sensors are very close and $a_{ij}\to \;0$ as the distance increases or exceeds ϵ, reflecting the inverse relationship between the interaction strength and the distance. The downstream adjacency matrix is defined as the transpose of the upstream adjacency matrix, i.e., ${A^{\prime }=A}^{T}$, allowing for reverse dependency modeling.

The spatiotemporal traffic state is represented by graph signals $X\in R^{n\times p}$, with *n* being the total number of spatial nodes (observed and unobserved) and *p* being the number of features per node.

### 3.2. Treating Traffic State Estimation as Kriging

This paper formulates network-wide TSE as a graph-based kriging problem, extending spatial interpolation techniques to infer traffic states at unobserved locations. A GNN-based kriging framework was adopted because GNNs naturally handle graph-structured data and capture complex dependencies through message passing. Our architecture explicitly leverages these capabilities to reconstruct traffic states from sparse sensor data, aligning with the principles of spatial interpolation.

Figure 1 illustrates the working mechanism of the framework. We modeled the entire road network as a graph, where nodes represent sensor locations. In reality, only a sparse subset of nodes equipped with sensors was observed. To enable our model to learn the generalizable patterns of reconstructing the complete field from partial observations, we employed a random masking strategy during training. This involved randomly treating different subsets of nodes in the full training data and masking out the rest, thereby simulating countless possible sparse sensor configurations. The model learned to propagate information from these randomly observed nodes to estimate the states of the masked nodes. After training, the model can be directly applied to estimate the traffic states of any unobserved locations in this road network, even if they are not in the training dataset.

Formally, we frame TSE as a regression problem where a model  f predicts traffic states $\hat{X}\;$ using input features X and graph G, as shown in Equation (2).

(2) $$\hat{X}=f\left(X,G;\theta \right)$$

where $\hat{X}$ denotes the estimated values and *X* incorporates the available input features, and θ represents the model parameters. In our implementation, the historical speed data and network geometry serve as inputs, while the ground truth speeds are used as training targets.

During deployment, the trained estimator ingests streaming data from arbitrary sensor locations and predicts the traffic states across the entire network. This inductive capability demonstrates that TSE can be effectively treated as a kriging problem that is solvable with GNNs.

## 4. Methodology

In this section, we propose a novel **p**hysics-**g**uided **s**patio**t**emporal **g**raph **c**onvolutional **n**etwork (PGSTGCN) model. The core of the PGSTGCN lies in utilizing both spatial correlations among nodes and temporal dependencies arising from the propagation of traffic waves.

**Figure 2:**
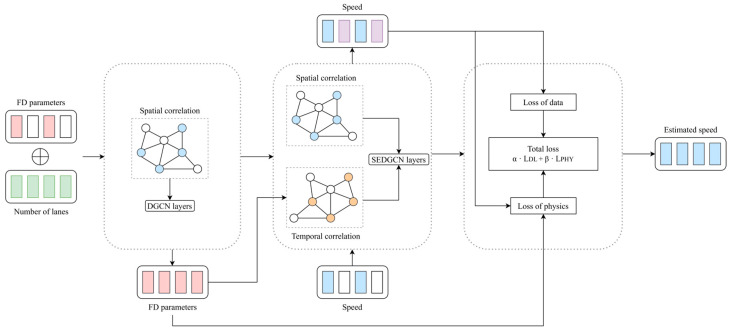
Overall architecture of the proposed PGSTGCN model.

Figure 2 shows the overall architecture of the PGSTGCN. It consists of three core components: (1) the estimation of the fundamental diagram (FD) parameters at unobserved locations, (2) a spatial and temporal information aggregation block that incorporates kinematic wave theory, and (3) the integration of physical constraints via the fundamental diagram. The model includes four stacked modified diffusion graph convolutional network (DGCN) layers and a physics-based loss function for speed estimation. The input consists of randomly masked speed data, and the PGSTGCN infers the complete traffic speeds across the network.

### 4.1. Wave-Informed Spatiotemporal Graph Construction

#### 4.1.1. Wave Speed Calculation

As introduced in Section 1, traffic wave propagation results in time-delayed correlations across nodes. To define these connections, we first calculated the wave speeds based on the FDs, as these speeds quantitatively determine the time it takes for a traffic state change to propagate from one node to another. FDs provide a well-established theoretical framework for characterizing the functional relationships among the traffic flow, density, and speed on road segments [44]. Wave speeds are given by the slopes of the FD curve. For observed nodes, the FD parameters are directly fitted to the data, but this is infeasible for unobserved nodes due to data scarcity.

Therefore, we adopted the DGCN model [43] to estimate the FD parameters for unobserved nodes, since the traffic network was modeled as a directed graph. DGCNs are widely applied to spatial interpolation by modeling traffic feature propagation as a finite diffusion process, enabling convolution on directional graphs. For an input graph signal matrix $X\;\in \;R^{N\times C_{m}}$, the DGCN operator can be generalized as shown in Equation (3):

(3) $$\hat{Y}=\left.\sum_{k=0}^{K}\left(A_{f}^{k}XW_{f}^{k}+A_{b}^{k}XW_{b}^{k}\right)\right.$$

where $A_{f}^{k},\;A_{b}^{k}$ are the forward and backward transition matrices at the *kth* diffusion step; *K* is the total number of diffusion steps; and $W_{f}^{k}\;,W_{b}^{k}\in \;R^{C_{in}\times \;C_{out}\;}$ are learnable parameters. This formulation captures the simultaneous influence of both inflows and outflows on the node states. The bidirectional transition matrices were derived from row-normalized adjacency matrices as follows:

(4) $$A_{f}=\frac{A}{rowsum\left(A\right)},\;A_{b}=\frac{A^{\prime }}{rowsum\left(A^{\prime }\right)}$$

This normalization ensures that the sum of the edge weights equals one, defining the diffusion probabilities between connected nodes.

The DGCN model, by exploiting directional node relationships among nodes, provides an ideal solution for FD parameter estimation. The FD parameters derived through nonlinear least-squares fitting serve as training labels for the DGCN. Taking the s-shaped three-parameter (S3) traffic flow model [45] as an example, where the precise speed–density relationship is unknown, the S3 model characterizes this relationship as a nonlinear function:

(5) $$v=\frac{v_{f}}{{\left[1+{\left(\frac{k}{k_{c}}\right)}^{m}\right]}^{\frac{2}{m}}}$$

where $v_{f}$ is the free-flow speed, $k_{c}$ is the critical density corresponding to maximum flow, and m is a shape parameter.

Of the three parameters in the S3 model, the free-flow speed $v_{f}$ and critical density $k_{c}$ have direct physical interpretations. The third parameter, the maximum flow inertia coefficient m, governs the shape and curvature of the fundamental diagram across different density regimes. Notably, it controls the nonlinear transition around the critical density and the slope of the congested branch $k>k_{c}$. While, for certain calibrated values of m, the flow–density relationship in congestion can exhibit an extended near-linear segment, the function remains fundamentally smooth and nonlinear. This is characterized by a variable backward wave speed and the presence of an inflection point in the flow–density curve, allowing the model to represent nuanced transitions between different congestion states more effectively than a piecewise or strictly linear approximation.

Our objective was to estimate these three parameters for each unobserved node. Since road capacity is affected by the lane number, which influences the free-flow velocity and critical density, the lane count was incorporated as an additional input feature alongside the parameters of neighboring nodes.

**Figure 3:**
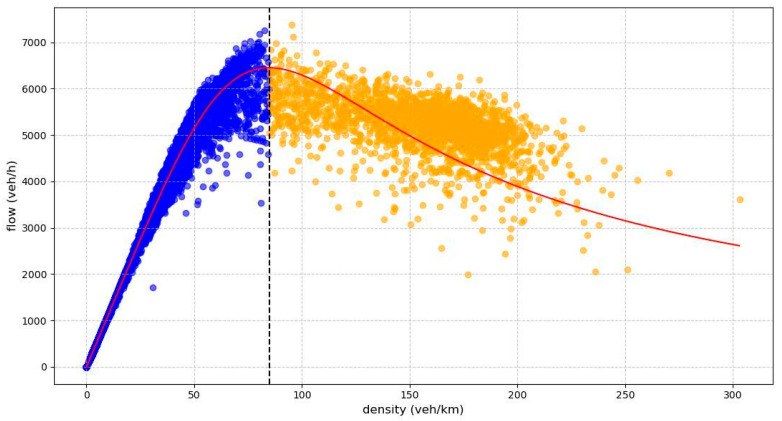
The two states of traffic flow. The red solid line represents the fitted curve. A black vertical dashed line divides the diagram into two regimes: free flow (left, blue dots) and congestion (right, orange dots).

Based on the wave propagation physics, the characteristic wave speeds are derived from the estimated FD parameters. According to the shape of the FD (Figure 3), traffic states are classified into two distinct phases: free-flow and congested. This classification allows us to compute the local wave speed at each sampled point on the fundamental diagram using Equation (6):

(6) $$c=\frac{q_{1}-q_{2}}{k_{1}-k_{2}}$$

To obtain representative wave speeds for each phase in a robust manner, we uniformly sampled n data points from the free-flow and congested branches of the fundamental diagram. The wave speed for each state was then approximated by averaging the derivatives across these points, as defined in Equation (7):

(7) $$c=\frac{1}{n}\sum_{i=1}^{n}\frac{\Delta q}{\Delta k}\left|\begin{array}{c} i \end{array}\right.\;$$

This procedure yielded two key wave speeds: the forward kinematic wave speed $c_{v}$ under free-flow conditions, and the backward shockwave speed $c_{w}$ under congested conditions. These wave speeds jointly define the spatial–temporal influence domain of traffic perturbations and provide a theoretical basis for constructing the directed adjacency matrix in subsequent sections.

#### 4.1.2. Spatiotemporal Graph Representation

While spatial graphs effectively capture correlations across nodes at the same time, they neglect dynamic dependencies across time. Prior studies have shown that combining temporal models with GNNs effectively addresses this issue [27,29], with GNNs learning spatial dependencies and temporal models capturing sequential patterns. However, such hybrids infer spatiotemporal relationships implicitly from data, which may result in spurious correlations and a lack of explicit physical causality. Temporal graphs can model these time-delayed correlations across nodes. Li and Zhu proposed the construction of temporal graphs from node-specific flow sequences [46]. However, this approach is limited for TSE due to the partial observability across networks.

In contrast, kinematic wave theory provides a physically grounded framework for characterizing these spatiotemporal evolutions [41]. These traffic dynamics are jointly governed by these two wave types [47,48]. Wave propagation exhibits key physical properties. First, hyperbolic propagation constrains traffic waves to finite speeds bounded by kinematic properties, generally not exceeding the free-flow limit. This governs the spatial extent to which perturbations spread over time. Second, driver response asymmetry implies that drivers react with latency to downstream events (e.g., deceleration), but are largely unaffected by upstream conditions. Together, these properties create a distinctly anisotropic spatiotemporal structure in traffic state transitions [49]. Disturbances in traffic flow form two distinct types of waves: forward-moving waves that travel with the traffic, and backward-moving shock waves that propagate against it. Consequently, the domain of influence for any perturbations is bounded by two characteristic velocities: the forward wave speed $c_{v}$ in free flow and the backward shock wave speed $c_{w}\;$ in congestion.

**Figure 4:**
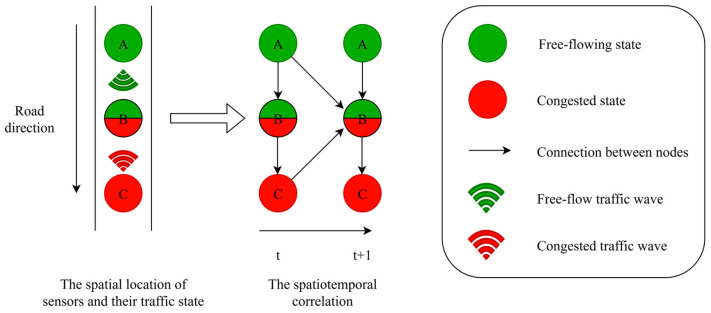
Construction of spatiotemporal graph.

To formalize this, let $X_{t}\in R^{n\times p^{\prime }}$ denote the state matrix at time step *t*, where *n* is the number of nodes and *p*^′^ is the feature count per node. For a preceding timestep τ, we define state matrix $X_{\tau }$. The causal constraint τ < *t* ensures that subsequent states cannot influence prior states. The causal influence from τ to *t* is encoded in bidirectional state transition matrices $A_{\tau }$ and ${A_{\tau }}^{\prime }$, where each element $A_{\tau }(i,\;j)$ or ${A_{\tau }}^{\prime }(i,\;j)$ quantifies the correlation between node i at time step  t and node  j at time step τ (illustrated in Figure 4). We consider that nodes traversed by relevant waves within a time interval ∆t are correlated with the state at node $v_{i}$. Specifically, free-flow waves propagate forward from node $v_{i}$ at a speed up to $c_{v}$, influencing a downstream region $I_{free}^{i}$ during ∆t. Conversely, shock waves propagate backward from $v_{i}$ at speed $c_{w}$, affecting an upstream region $I_{cong}^{i}$ in the same time interval. Thus, the estimated state at $v_{i}\;$ depends on observations within the union $I_{free}^{i}\cup I_{cong}^{i}$. We incorporated these wave-based mechanics into the adjacency matrix, as defined in Equation (8):

(8) $$a_{ij}=\left\{\begin{array}{c} \exp\left(-{\left(\frac{dist\left(v_{i},v_{j}\right)/|c|}{\delta \prime }\right)}^{2}\right),\;if\;dist\left(v_{i},\;v_{j}\right)\leq \left|c\right|\cdot (t-\tau ),\\ 0,\;otherwise \end{array}\right.$$

where c denotes $c_{v}$ for the free-flow adjacency matrix or $c_{w}$ for the congested matrix, and $\delta^{\prime }$ is the standard deviation of $dist(v_{i},v_{j})/|c|$.

In order to capture more comprehensive relationships, we incorporated spatial and temporal graphs into a novel united graph, which simplified the learning of spatiotemporal dependencies. The fusion graph makes GNN lightweight and enables highly efficient computations through straightforward matrix operations, eliminating the need for complex spectral filtering or heavy parameterization. It inherently encodes three critical types of correlations through simple and fast matrix multiplications: (1) direct spatial neighbors, (2) time-delayed correlations, and (3) the node’s own historical and future states along the time axis.

We defined the fused graph as $\tilde{G}=(\tilde{V},\;\tilde{E},\tilde{A})$. The node set was defined as $V=\{v^{1},v^{2},\;\ldots ,v^{N}\}$, and $v^{t}$ denotes the physical nodes at time step t. The edge set $\tilde{E}=\left\{{\tilde{e}}_{ij}\right\}$ denotes both spatial connections and temporal state transitions across time steps. $\tilde{A}\in R^{\left(N\times T\right)\times (N\times T)}$ quantifies the correlation strength between all such spatiotemporal node pairs. On this graph, the input traffic data are represented as graph signals $X=R^{(n\times h)\times p}$, and the goal of the GNN-based model is to learn a function to reconstruct the complete matrix $Y\;\in \;R^{\left(n\times h\right)\times p}$. The forward and backward spatiotemporal adjacency matrices are constructed by splicing the spatial relationships and temporal state transition matrices, as shown in Equation (9):

(9) $${\tilde{A}}_{f}=\left[\begin{array}{ccc} A_{1,1}^{f} & \ldots & 0\\ \vdots & \ddots & \vdots \\ A_{\left(t,1\right)}^{cong} & \ldots & A_{t,t}^{f} \end{array}\right],\;{\tilde{A}}_{b}=\left[\begin{array}{ccc} A_{1,1}^{b} & \ldots & 0\\ \vdots & \ddots & \vdots \\ A_{\left(t,1\right)}^{free} & \ldots & A_{t,t}^{b} \end{array}\right]$$

### 4.2. Architecture of GNN Framework

For spatiotemporal modeling, the DGCN model, previously demonstrated as an effective interpolation model in Section 4.1, provides an ideal framework by exploiting directional node relationships. Different from FD parameter estimation, the strength of bidirectional dependencies varies considerably across traffic conditions. The conventional DGCN model treats the upstream and downstream connections as fixed-weight adjacency relations, aggregating them uniformly using globally shared parameters. However, this formulation lacks adaptability to the time-varying nature of traffic states, limiting the capacity of the model to fit dynamic traffic scenarios.

**Figure 5:**
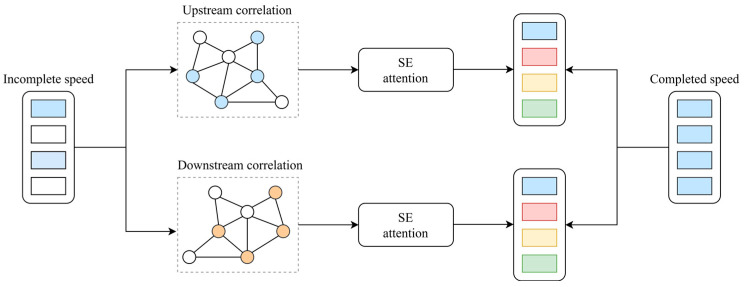
Mechanism of SEDGCN module.

To address this limitation, we incorporated a channel attention mechanism, specifically the squeeze-and-excitation (SE) module [50], to project bidirectional diffusion features into the channel dimension. The mechanism of the squeeze-and-excitation diffusion convolutional network (SEDGCN) is shown in Figure 5. This enhancement leads to a refined model termed the SEDGCN, which modifies the foundational DGCN architecture through SE-based attention. By learning attention weights conditioned on both time steps and traffic states, the SEDGCN dynamically fuses forward and backward diffusion features. This enables the model to adapt to time-varying dependencies, thereby improving the TSE accuracy.

The module processed the input tensor $X\in R^{B\times N\times T}$ through a hierarchical attention pipeline designed to capture dynamic spatiotemporal dependencies. First, truncated mean pooling, as shown in Equation (10), compresses the temporal dynamics across the entire time dimension T, summarizing the behavior of each node channel while remaining robust to outliers. This operation generates a compact channel descriptor vector z, which encapsulates the global temporal signature of each feature channel across all nodes.

(10) $$z=\frac{1}{T}\sum_{t=1}^{T}x(t)$$

The descriptor z is then fed into a lightweight, parameter-efficient neural network, typically composed of two convolutional layers, as shown in Equation (11). This network learns nonlinear interactions among feature channels and outputs a channel-wise excitation vector g, where each element represents the importance of the corresponding upstream information channel under the current hierarchical and temporal context.

(11) $$g=\sigma \left(W_{2}\cdot ReLU\left(W_{1}\cdot z+b_{1}\right)+b_{2}\right)$$

where g is the weight of the upstream information; $W_{1},\;W_{2}$ are the learnable parameters; and $\sigma \left(\cdot \right)$ is an activation function, such as the sigmoid function.

The excitation vector g serves as an adaptive gating signal. It is directly applied in the original DGCN layers to modulate the contribution of upstream information across different channels during spatial aggregation, as expressed in Equation (12).

(12) $$\begin{array}{c} {\hat{H}}_{t}^{1}=\left.\sum_{k=0}^{K}\left(g⨀{\tilde{A}}_{f}^{k}\tilde{X}W_{f}^{k}+(1-g)⨀{\tilde{A}}_{b}^{k}\tilde{X}W_{b}^{k}\right)\right.,l=0\\ {\hat{H}}_{t}^{l+1}=\sigma \left.\left(\sum_{k=0}^{K}\left(g⨀{\tilde{A}}_{f}^{k}\tilde{X}W_{f}^{k}+(1-g)⨀{\tilde{A}}_{b}^{k}\tilde{X}W_{b}^{k}\right)\right)\right.,l\geq 1 \end{array}$$

where l is the number of DGCN layers; σ(·) denotes the activation function, such as the ReLU function; and ⨀ is the Hadamard product. Since the unsampled nodes cannot provide useful information for their neighborhood, we stacked several DGCN layers to capture more abundant representations and output the estimated results.

### 4.3. Loss-Function-Integrated Fundamental Diagram

Based on the formulation of network-wide traffic state estimation as a specialized kriging task, our objective was to reconstruct manually masked subgraphs and generalize to unsampled locations during testing. To promote a universal message-passing mechanism applicable to all nodes, we defined the loss function as the total reconstruction error over both the observable and unobservable nodes. Conventional loss functions in related studies are typically data-driven and based on the estimation error, expressed in the form of Equation (13):

(13) $$L_{DL}=\frac{1}{n}\sum_{i=1}^{n}\left.{\left({\hat{X}}_{i}-X_{i}\right.)}^{2}\;\right.$$

where ${\hat{X}}_{i}$ denotes the estimated values and $X_{i}$ is the true value.

However, relying exclusively on such a data-driven loss $L_{DL}$ has notable limitations. It may lead to overfitting to spurious patterns in the training data. More critically, for physical systems like traffic, a purely statistical model can generate estimates that violate fundamental flow principles, undermining the reliability and interpretability.

To alleviate this problem, we introduced a physic-based loss term $L_{PHY}$ grounded in the FD, which has been shown to be effective [37,40]. The FD graphically represents relationships among the flow q, density k, and speed v. Incorporating it as a soft constraint provides several key advantages for TSE. Firstly, it explicitly penalizes estimates that violate the macroscopic FD relationships based on the consistency of different variables, ensuring that the outputs are physically plausible. Next, by privileging solutions that adhere to physical laws, the model becomes less sensitive to measurement noise and outliers in the input data, as these often manifest as FD violations. The specific FD equation adopted in this study is given by Equation (11).

A key challenge is that empirical traffic data seldom conform perfectly to the theoretical FD curve, often scattering above and below it due to measurement noise, non-stationarity, or unmodeled factors. Imposing the FD as a hard constraint could force the model to ignore legitimate data fluctuations, potentially degrading the estimation accuracy and generalization.

Therefore, to robustly handle the deviation between observed data points and the theoretical FD curve, we adopted the Huber loss for LPHY. The Huber loss, commonly used in robust regression, combines the squared error for small deviations and the absolute error for larger ones, defined in Equation (14):

(14) $$\begin{array}{c} L_{PHY}=\frac{1}{n}\sum_{i=1}^{n}l_{PHY}\\ l_{PHY}=\left\{\begin{array}{c} \frac{1}{2}{\left(\hat{X_{i}}-{\tilde{X}}_{i}\right)}^{2},if\;\left|\hat{X_{i}}-{\tilde{X}}_{i}\right|\leq \delta \\ \delta \left|\hat{X_{i}}-{\tilde{X}}_{i}\right|-\frac{1}{2}\delta^{2},if\;\left|\hat{X_{i}}-{\tilde{X}}_{i}\right|\geq \delta \end{array}\right. \end{array}$$

where ${\tilde{X}}_{i}$ is the theoretical value computed by the fundamental diagram equation, ${\hat{X}}_{i}$ denotes the estimated value of the model, and δ is a hyperparameter.

The Huber loss offers a superior robustness to outliers compared to the squared error. For deviations beyond the threshold δ, it applies a linear penalty (absolute error), thereby reducing the influence of outliers while maintaining differentiability for small errors. This is ideal for gradient-based optimization in regression tasks like TSE, where significant deviations may occur.

In summary, the composite loss function is defined as the weighted sum of the data-driven loss and the physics-based loss, as shown in Equation (15):

(15) $$L\;=\;\alpha L_{DL}\;+\;\beta L_{PHY}$$

where L is the total loss and α and β denote the weights of the two loss items.

### 4.4. Model Implementations

Consistent with the inductive learning framework introduced by Wu et al. [25], our training strategy employed a stochastic subgraph masking and reconstruction procedure (detailed in Section 3.2). Specifically, the model was trained on partially masked graph signals as inputs and learned to reconstruct the complete graph signals as outputs. This configuration enables the PGSTGCN to effectively learn representations from incomplete graph structures while simultaneously acquiring the ability to generalize to unobserved nodes absent from the training graph. The formal training algorithm is provided in Algorithm 1. Critically, this inductive paradigm supports real-time traffic state kriging that utilizes instantaneous sensor measurements, offering a distinct advantage over tensor completion methods in dynamic, real-time scenarios.

**Algorithm** **1.** PGSTGCN training procedureInput:

$\mathrm{Training}\;\mathrm{speed}\;\mathrm{data}:\;X_{s}\in R^{n_{o}\times T}$

Forward adjacency matrix ${\tilde{A}}_{f}$, backward adjacency matrix ${\tilde{A}}_{b}$Time propagation steps HHidden dimension ZDiffusion steps KBatch size BNumber of batches *N_b_*Masked nodes per sample *n_m_*Max iterations *I*Output:Trained model *model*Initialize the model as $model^{0}=PGSTGCN(H,Z,K)$for iteration *i* = 1 to *I* do          for batch *n* = 1 to *I* to *N_b_* do                    Generate batch indices:                    $T_{r}\leftarrow RandomSample(0,T-H|,B)$                    Initialize $X_{v}\in R^{B\times n_{0}\times H}\leftarrow 0,\;M\in {\left[0,1\right]}^{B\times n_{0}\times L}\leftarrow 1$                    for sample *b* = 1 to B do                               Extract time sequences:                               $X_{v}[b,:,:]\leftarrow X_{s}[:,T_{r}\left[b\right]:\;T_{r}\left[b\right]+H]$                               Generate node mask:                               $M_{r}\leftarrow RandomSample([1,n_{o}],n_{m})$                               $M[b,M_{r},:]\leftarrow 0$                    end for                    Prepare masked input:                    $X_{v}^{masked}\leftarrow M⨀X_{v}$                    Forward pass                    ${\hat{X}}_{v}\leftarrow model^{i-1}(X_{v}^{masked},\;{\tilde{A}}_{f},\;{\tilde{A}}_{b})$                    Compute loss                    $L\leftarrow Loss\left({\hat{X}}_{v},\;X_{v},\;M\right)computed\;by\;\mathrm{Equation}\;(15)$                    Backward pass and update                    Compute ∇L w.r.t parameters                    Update parameters with Adam          end forend forReturn $model^{1}$

## 5. Numerical Experiments

This section presents the experiments and results on a public highway dataset to validate the effectiveness of the proposed model.

### 5.1. Dataset Description and Experimental Setup

#### 5.1.1. Highway Traffic Dataset

**Figure 6:**
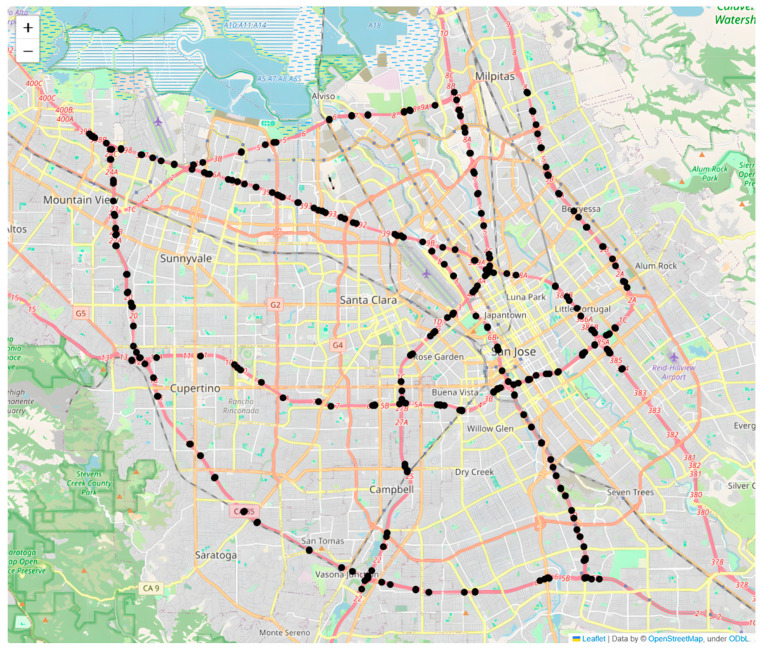
Sensor distribution of the PeMS-Bay dataset.

To evaluate the performance of the proposed model, we conducted case studies using PeMS-Bay traffic data collected from the Performance Measurement System of California (PeMS) in real time by over 39,000 sensor stations, deployed across the major metropolitan areas of the California state highway system. This traffic dataset is aggregated into 5 min intervals from 30 s data samples. It contains 325 sensors in the Bay Area and 3 months of data were collected, ranging from January to March 2018 [28]. The distribution of the sensors is provided in Figure 6. The key attributes of the traffic observations and geographic information are also provided.

#### 5.1.2. Configurations

The hardware platform on which the experiments were carried out was the Windows 11 platform with an Intel Core i5-12500H CPU and an NVIDA GeForce RTX 3050 GPU. The software environment consisted of Python 3.12.0 and Pytorch 2.3.0.

#### 5.1.3. Evaluation Metrics

To evaluate the model performance, we employed three quantitative metrics: the mean absolute error (MAE), the root mean squared error (RMSE), and the mean absolute percentage error (MAPE).

The MAE measures the average magnitude of absolute errors between the ground truth and the estimated values, defined in Equation (16):

(16) $$MAE=\frac{1}{N}\sum_{n=1}^{N}\left|{\hat{X}}_{n}-X_{n}\right|$$

where ${\hat{X}}_{n}$ and $X_{n}$ denote the estimated value and the ground truth, respectively, and *N* is the total number of test samples.

The RMSE quantifies the square root of the mean squared error between the ground truth and the estimated values, calculated using Equation (17):

(17) $$RMSE=\sqrt{\frac{1}{N}\sum_{n=1}^{N}{\left({\hat{X}}_{n}-X_{n}\right)}^{2}}$$

The MAPE measures the mean absolute percentage error relative to the ground truth, defined in Equation (18):

(18) $$MAPE=\frac{1}{N}\sum_{n=1}^{N}\left|\frac{{\hat{X}}_{n}-X_{n}}{X_{n}}\right|$$

Crucially, different from the training objective (which targets both observable and unobservable nodes), our evaluation focused specifically on the performance at unobserved sensor locations within the road network.

#### 5.1.4. Baseline Models and Experimental Setup

To validate the model performance, we conducted comparative evaluations against the following baselines: ordinary kriging, K-nearest neighborhoods (KNN), a graph convolutional network (GCN), Bayesian Gaussian CANDECOMP/PARAFAC (BGCP) tensor decomposition [51], inductive graph neural network kriging (IGNNK) [25], and Laplacian-enhanced low-rank tensor completion (LETC) [52]. Brief methodological descriptions are provided.

**Ordinary Kriging**: This is a geostatistical interpolation technique that estimates values at unmeasured locations by accounting for spatial correlations among data points and assumes a constant, but unknown, mean.

**KNN**: This is a spatial kriging benchmark that estimates unobserved states via the arithmetic mean of neighboring observations.

**GCN**: This is a GNN architecture designed for graph-structured data that updates node representations by aggregating features from adjacent nodes.

**BGCP**: This is a high-order Bayesian probabilistic tensor factorization model for spatiotemporal traffic data imputation.

**IGNNK**: This is an inductive graph neural network for traffic speed kriging that utilizes diffusion convolutional operations.

**LETC**: This is a spatiotemporal tensor completion model that incorporates Laplacian regularization within a unified tensor nuclear norm minimization framework to capture spatiotemporal correlations and low-rank structure.

The experimental settings are specified as follows. The nodes were randomly partitioned into observed sensors and unsampled locations. For the deep learning baselines, the temporal data were partitioned into training (70%), validation (15%), and testing (15%) sets. The key hyperparameters for the PGSTGCN were as follows. For the DGCN layer, the diffusion step was set to 1, the hidden dimension was set to 80, and the number of DGCN layers was 4. In the training stage, the input batch size was 32, and we chose the Adam optimizer with a fixed learning rate of 1e-3. The initial evaluation compared all models at a 70% sensor coverage rate, i.e., 159 sensors were randomly selected as observed locations used for model training, 69 sensors were used for validation, and the remaining 97 sensors were used for evaluation during testing.

### 5.2. Estimation Results and Model Comparison

#### 5.2.1. Parameter Determination

**Figure 7:**
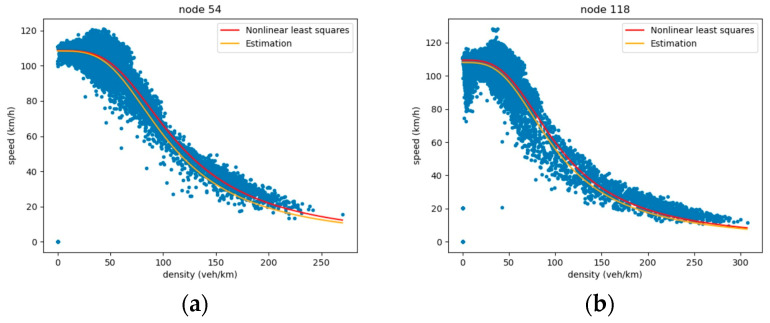
Snapshots of estimated values and ground truth at some specific unobserved nodes. (**a**) The result of node 54. (**b**) The result of node 118.

This section presents the FD parameter estimation results across varying sensor missing rates. We defined actual traffic speed measurements as the ground truth and values derived from FD curves as the estimated values. Table 1 summarizes the FD parameter calibration results, indicating a satisfactory overall performance. As illustrated in Figure 7, the calibrated FD curves showed reasonable overall agreement with the data and successfully captured the trend. The performance at a 70% coverage rate was lower than in other scenarios. The main reasons for this may be the randomness in the training data splits and the stochastic nature of model training. The overall trend still supports the benefit of increased data coverage. Consequently, these validated FD parameters are suitable for enforcing physical constraints, including wave speed computation and physics-guided loss function terms.

#### 5.2.2. Speed Estimation Results

**Figure 8:**
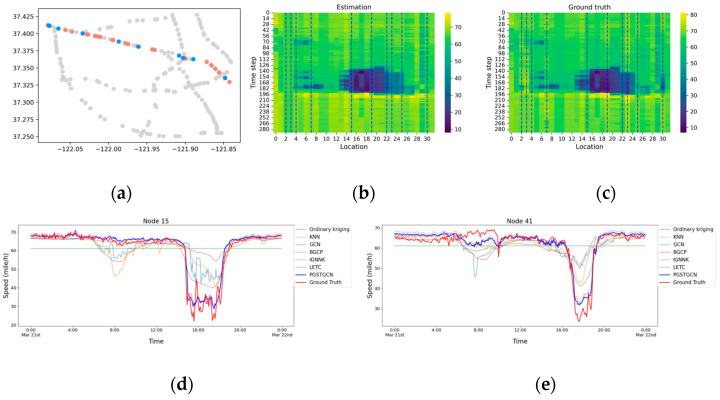

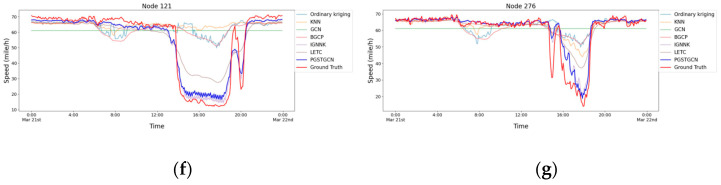
Comparison of the estimated and true values of the speed and snapshots at specific nodes. (**a**) The nodes in the heatmaps. (**b**) The heatmap of the estimation. (**c**) The heatmap of the ground truth. (**d**) The result of node 15. (**e**) The result of node 41. (**f**) The result of node 121. (**g**) The result of node 276.

We evaluated the proposed PGSTGCN model against baseline methods under a 70% sensor coverage scenario to demonstrate its superiority. The experimental results are presented in Table 2 and Figure 8. Table 2 quantitatively compares the speed estimation errors of the PGSTGCN and the baselines. Given the large spatiotemporal intervals between the sensors and observations, we exclusively considered traffic wave propagation within a single time step (T = 2). Figure 8 visually contrasts the PGSTGCN estimates against the ground truth at several randomly selected nodes within a spatiotemporal domain.

According to the results, we drew the following conclusions:

(1) High Performance: As shown in Table 2, all the evaluated models achieved low estimation errors, with our model attaining the best overall performance. To further illustrate the effectiveness of the proposed model, Figure 8 presents a detailed comparison of the estimated and ground truth speeds over one day (288 time points) at four representative nodes. We chose some nodes in the same direction, where the red dots are observable and the blue dots are unobservable. The heatmaps reflect the phenomenon of shockwaves propagating on the road, and the blue dotted line represents the estimated nodes. The results show that the estimations of the proposed model (blue line) consistently tracked the ground truth (red line), accurately capturing both short-term fluctuations and overall daily trends. This demonstrates its powerful capability for TSE. An analysis of specific unobserved nodes revealed important patterns. Most models, including ours, closely approximated the ground truth speeds during periods of stable traffic flow. The performance diverged significantly during congestion-induced speed transitions. While our model maintained its accuracy, most baselines exhibited substantial deviations during these abrupt changes. Notably, the GCN model produced moderate error values, but failed to capture the underlying speed variation trend. In contrast, our model provided both accurate and physically plausible estimations across diverse traffic states.
(2) Robustness: Given the relative stability of freeway traffic flow and the potential for kinematic waves to propagate over considerable distances, we further evaluated the model robustness under different traffic wave diffusion steps. Table 3 reports the imputation accuracy of the PGSTGCN and baselines models in multi-step data recovery tasks (T = 2, 3). The PGSTGCN consistently demonstrated a superior performance, demonstrating its adaptability and reliability across varying temporal scales.

#### 5.2.3. Model Performances Under Lower Coverage Rates

**Figure 9:**
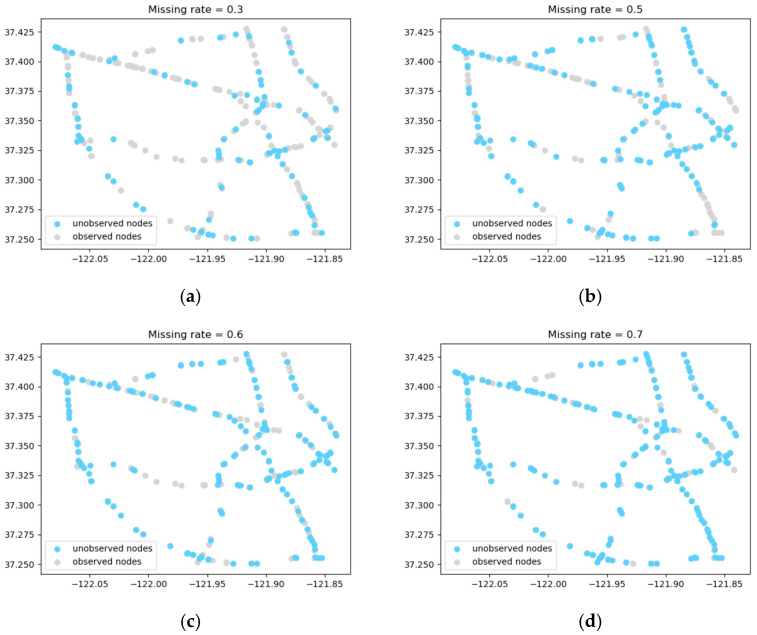
Sensor distribution at different coverage rates. (**a**) Coverage rate = 70%. (**b**) Coverage rate = 50%. (**c**) Coverage rate = 40%. (**d**) Coverage rate = 30%.

**Figure 10:**
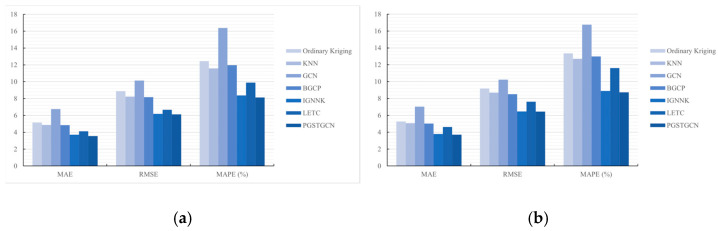

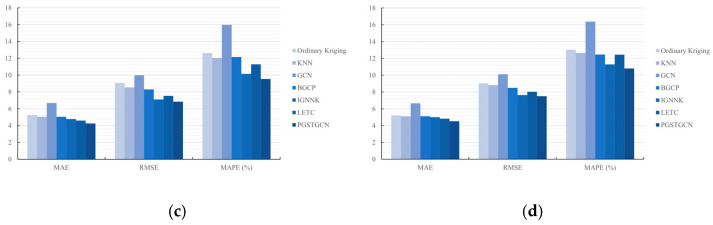
Model performance under different coverage rates. (**a**) Coverage rate = 70%. (**b**) Coverage rate = 50%. (**c**) Coverage rate = 40%. (**d**) Coverage rate = 30%.

To evaluate the model effectiveness under sparse sensor deployments, we conducted experiments at 50%, 40%, and 30% sensor coverage rates. Table 4 summarizes the quantitative evaluation metrics, while Figure 9 visualizes the spatial distribution of both the observed and unobserved sensors across different missing scenarios. The comparative model performance is further illustrated in Figure 10.

As anticipated, a reduced sensor coverage significantly deteriorated the model performance due to information scarcity. Notably, the PGSTGCN consistently outperformed the baseline models across all low-coverage scenarios, demonstrating a superior estimation accuracy. These results substantiate the robustness and practical utility of the PGSTGCN in data-sparse environments.

### 5.3. Ablation Studies

To quantify the contributions of each component in the proposed model, we conducted ablation studies at a 70% sensor coverage rate by systematically removing or modifying specific modules:

PGSTGCN w/o temporal correlation (TC): We replaced temporal correlations with only a static distance-based spatial graph.

PGSTGCN w/o spatial correlation (SC): We replaced spatial correlations with only a wave-informed temporal graph.

PGSTGCN w/o FD: We removed the FD physics loss term with the total loss function.

PGSTGCN w/o SE: We removed the SE attention mechanism from the DGCN model.

PGSTGCN w/o SEDGCN: We substituted the direction-aware diffusion convolution with symmetric spectral convolution.

As evidenced in Table 5, all the ablated variants exhibited a degraded performance compared to the full PGSTGCN, underscoring the necessity of each component. Removing either the spatial or temporal module led to a significant increase in the estimation error, confirming that the speed dynamics are governed by both spatial neighborhood interactions and temporal wave propagation. The ablation of the SE mechanism revealed the dynamic and time-varying nature of node dependencies, indicating that the influence from upstream and downstream nodes varies considerably across different stages. The SEDGCN ablation further demonstrated the importance of explicitly encoding direction-aware topology in the graph convolution for traffic networks.

Although the direct improvement in the overall metrics from the FD constraint was moderate, this physics-informed regularization effectively suppressed outlier predictions and enforced physical plausibility. By penalizing estimates that violate fundamental flow–density relationships, the FD loss term restricts the solution space to physically consistent outputs, enhancing the interpretability and stability of the model, particularly in data-sparce or extrapolation scenarios.

## 6. Conclusions and Future Directions

Acquiring high-resolution spatiotemporal traffic state data remains a significant challenge, largely due to the limited deployment of traffic detectors across highway networks. Sparse sensor coverage results in incomplete observations, which complicates real-time traffic management and control. Traditional model-driven and purely data-driven methods struggle under such conditions, either requiring extensive calibration or failing to capture the underlying physics of traffic dynamics. To address these limitations, this paper introduces a physics-guided spatiotemporal graph convolution network (PGSTGCN) that is characterized by its ability to account for both spatial dependence and temporal correlations, and it was specifically designed for traffic state estimation in scenarios with sparse sensor coverage.

The proposed framework explicitly incorporates the effects of kinematic wave propagation, which governs how traffic disturbances evolve and spread across road segments. To this end, we designed spatial and temporal dependency modules that jointly model structural similarities between sensor locations constrained by network topology, and an-isotropic temporal correlations induced by wave propagation. By embedding these physics-guided dependencies into a graph neural network, the PGSTGCN captures both local interactions and long-range causal relationships that conventional GNN–temporal hybrids often miss.

Extensive experiments were conducted on a real-world highway traffic speed dataset to evaluate the effectiveness of the PGSTGCN. The key findings were as follows:

(1) Superior performance under sparse observations: Compared with baseline deep learning and tensor-learning methods, the PGSTGCN consistently achieved a higher estimation accuracy across varying sensor coverage conditions, highlighting its robustness to data sparsity.
(2) Effectiveness of model components: Ablation studies confirmed the contribution of each module. The spatial and temporal graph modules accurately captured spatiotemporal dependencies, while the diffusion-based GCN enhanced estimation by leveraging both forward- and backward-propagating relationships in traffic flow.

Despite its strong performance, several limitations remain. First, the validation was confined to highway networks. The underlying assumptions of the model may not hold robustly in urban networks with traffic signals or during non-recurring incidents, limiting the model’s current applicability. Second, the spatiotemporal graph construction led to quadratic complexity growth with the time window, posing challenges for large-scale, real-time deployment. Third, the framework showed a varied effectiveness across traffic variables (e.g., less satisfactory for volume estimation) and would benefit from broader benchmarking against diverse model types, such as model-driven and hybrid methods, to precisely define its comparative advantages. Future research will therefore focus on reducing the computational burden, improving generalizability, and extending the model to broader traffic variables and multimodal datasets.

## Figures and Tables

**Table 1:** FD parameter estimation results under different coverage rates.

	70%	50%	40%	30%
MAE	2.83	2.27	2.38	2.61
RMSE	4.62	3.86	4.12	4.27
MAPE (%)	5.23	4.25	4.43	4.85

**Table 2:** Speed estimation results of different methods under 70% coverage rate.

	Ordinary Kriging	KNN	GCN	BGCP	IGNNK	LETC	PGSTGCN	Improve
MAE	5.15	4.88	6.75	4.86	3.72	4.12	**3.5** **5**	4.6%
RMSE	8.89	8.23	10.14	8.18	6. 19	6.68	**6.** **14**	0.8%
MAPE (%)	12.44	11.57	16.37	11.97	8.38	9.89	**8.** **14**	2.9%

The best results are bold-marked, while the second-best results are underlined. “Improve” indicates the percentage of the PGSTGCN’s relative performance improvement over the most competitive baseline.

**Table 3:** Speed estimation results for different time steps at 70% coverage rate.

	T = 2			T = 3		
	MAE	RMSE	MAPE	MAE	RMSE	MAPE
GCN	6.75	10.14	16.37	7.03	10.21	16.71
IGNNK	3.72	6.19	8.38	3.88	6.17	8.37
PGSTGCN	3.55	6.14	8.14	3.69	6.10	8.08

**Table 4:** Model comparison at lower coverage rates.

		Ordinary Kriging	KNN	GCN	BGCP	IGNNK	LETC	PGSTGCN	Improve
50%	MAE	5.27	5.07	7.03	5.04	3.79	4.62	**3.7** **2**	1.8%
	RMSE	9.18	8.70	10.24	8.51	6.47	7.63	**6.** **45**	0.3%
	MAPE	13.36	12.71	16.76	12.99	8.90	11.62	**8.7** **4**	1.8%
40%	MAE	5.25	5.04	6.69	5.05	4.77	4.59	**4.** **26**	7.2%
	RMSE	9.08	8.54	9.99	8.31	7.10	7.54	**6.** **84**	3.7%
	MAPE	12.61	12.04	15.98	12.16	10.14	11.27	**9.** **53**	6.0%
30%	MAE	5.21	5.12	6.65	5.12	5.02	4.82	**4.53**	6.0%
	RMSE	9.05	8.81	10.11	8.49	7. 6 5	8.03	**7.** **50**	2.0%
	MAPE	13.02	12.64	16.37	12.47	11. 28	12.45	**10.** **81**	2.4%

The best results are bold-marked, while the second-best results are underlined.

**Table 5:** The results of ablation studies.

	PGSTGCN	PGSTGCN w/o TC	PGSTGCN w/o SC	PGSTGCN w/o FD	PGSTGCN w/o SE	PGSTGCN w/o SEDGCN
MAE	**3.5** **5**	3.87	3.95	3.58	3.61	6.27
RMSE	**6.** **14**	6.50	6.64	6.16	6.21	10.21
MAPE (%)	**8.** **14**	8.66	9.06	8.18	8.32	15.90

Best results are bold-marked.

## Data Availability

The data that support the findings of this study are openly available in the Performance Measurement System (PeMS) at https://pems.dot.ca.gov.
